# Construction of a Novel Signature and Prediction of the Immune Landscape in Soft Tissue Sarcomas Based on N6-Methylandenosine-Related LncRNAs

**DOI:** 10.3389/fmolb.2021.715764

**Published:** 2021-10-15

**Authors:** Li Zhang, Xianzhe Tang, Jia Wan, Xianghong Zhang, Tao Zheng, Zhengjun Lin, Tang Liu

**Affiliations:** ^1^ Department of Orthopedics, The Second Xiangya Hospital of Central South University, Changsha, China; ^2^ Department of Endocrinology, The Fifth Central Hospital of Tianjin, Tianjin, China; ^3^ Department of Orthopedics, Chenzhou No. 1 People's Hospital, Chenzhou, China; ^4^ Xiangya School of Medicine, Central South University, Changsha, China

**Keywords:** soft tissue sarcomas, N6-methyladenosine methylation, long noncoding RNAs, immune microenvironment, prognostic signature

## Abstract

**Background:** N6-methylandenosine-related long non-coding RNAs (m6A-related lncRNAs) are critically involved in cancer development. However, the roles and clinical significance of m6A-related lncRNAs in soft tissue sarcomas (STS) are inconclusive, thereby warranting further investigations.

**Methods:** Transcriptome profiling data were extracted from The Cancer Genome Atlas (TCGA) database and Genotype-Tissue Expression (GTEx). Consensus clustering was employed to divide patients into clusters and Kaplan–Meier analysis was used to explore the prognostic differences between the subgroups. Gene set enrichment analysis (GSEA) was conducted to identify the biological processes and signaling pathways associated with m6A-Related lncRNAs. Finally, patients were randomly divided into training and validation cohorts, and least absolute shrinkage and selection operator (LASSO) Cox regression was conducted to establish the m6A-related lncRNA-based risk signature.

**Results:** A total of 259 STS patients from TCGA-SARC dataset were enrolled in our study. Thirteen m6A-Related lncRNAs were identified to be closely related to the prognosis of STS patients. Patients were divided into two clusters, and patients in cluster 2 had a better overall survival (OS) than those in cluster 1. Patients in different clusters also showed differences in immune scores, infiltrating immune cells, and immune checkpoint expression. Patients were further classified into high-risk and low-risk subgroups according to risk scores, and high-risk patients were found to have a worse prognosis. The receiver operating characteristic (ROC) curve indicated that the risk signature displayed excellent performance at predicting the prognosis of patients with STS. Further, the risk signature was remarkably connected with the immune microenvironment and chemosensitivity in STS.

**Conclusion:** Our study demonstrated that m6A-related lncRNAs were significantly associated with prognosis and tumor immune microenvironment and could function as independent prognosis-specific predictors in STS, thereby providing novel insights into the roles of m6A-related lncRNAs in STS.

## Introduction

Soft tissue sarcomas (STS) are a heterogeneous group of various rare tumors of mesenchymal origin that most frequently occur in the extremities (Bourcier et al., 2019). Despite recent progress in STS treatment, including surgery, chemotherapy, and radiotherapy, the survival rates of patients with advanced STS still need to be improved. The 5-year survival rate of advanced STS patients is as low as 27.2% (Kim et al., 2019). Further, approximately 50% of STS patients would eventually develop distant metastases, which remains as a major cause of death and poses an obstacle to effective treatment (Italiano et al., 2011). There are more than 70 subtypes of STS with distinct biological phenotypes, molecular aberrations, and clinical outcomes. Accordingly, it is difficult to predict the prognosis of patients with STS (Jo and Fletcher, 2014). Currently, there is an urgent need to identify novel biomarkers to predict prognosis and evaluate the risks for STS patients.

N6-methyladenosine (m6A) was first discovered in the 1970s and is considered the most abundant form of internal mRNA modification (Adams and Cory, 1975; Wang et al., 2020). m6A modifications are modulated by various proteins that are classified into three categories: m6A writers, erasers, and readers (Yang et al., 2018). m6A writers, including the methyltransferase-like (METTL) family (METTL3/5/14/16), RNA-binding motif protein 15/15B (RBM15/15B), WTAP, HAKAI, ZC3H13, and KIAA1429, can execute the m6A methylation process (Meyer and Jaffrey, 2017; Yang et al., 2018). m6A modification is also a dynamic and reversible process, which can be demethylated by the m6A erasers, FTO and ALKBH5 (Song et al., 2019). m6A readers consist of the YTH domain family (YTHDF) family (YTHDF1/2/3), YTH domain-containing (YTHDC) family (YTHDC1/2), IGFBP family proteins, HNRNPC, FMR1, and EIF3A, which are responsible for decoding m6A methylation and recruiting downstream functional complexes (Zaccara et al., 2019). Based on emerging evidence, m6A modifications play critical roles in cancer initiation and progression in various cancer types (Muthusamy, 2020; Zhao et al., 2020). For instance, WTAP, which is highly expressed in osteosarcoma tissues and linked with the worse prognosis of osteosarcoma patients, was found to potentially promote osteosarcoma progression by inhibiting HMBOX1 in an m6A-dependent manner *in vitro* and *in vivo* (Chen et al., 2020). However, only few studies have investigated the potential roles of m6A modification in STS, and the regulatory roles of m6A modification in STS. Moreover, the detailed mechanisms have not been extensively assessed.

LncRNAs comprise a group of noncoding RNAs (ncRNAs) longer than 200 nucleotides that lack protein-coding potential (Evans et al., 2016). Multiple studies have demonstrated that several lncRNAs are dysregulated during cancer development and are critically involved in diverse cellular processes, such as cellular proliferation, apoptosis, and chemoresistance in human malignancies (Cheng et al., 2019; Mishra et al., 2019). For instance, the overexpression of linc00423, a downregulated lncRNA in liposarcoma patients, has been found to inhibit liposarcoma cell proliferation and colony formation by suppressing the MAPK signaling pathway via direct binding with NFATC3 *in vivo* and *in vitro* (Zhang et al., 2019). Notably, the interactions between m6A modifications and lncRNAs in cancer development have been investigated in several recent studies (Ma et al., 2019). m6A modifications of lncRNAs can modulate the localization, transport, and cleavage of lncRNAs. Notably, lncRNAs also play a regulatory role in m6A modifications, and the crosstalk between m6A modifications and lncRNAs can play critical roles in cancer progression (Coker et al., 2019). For instance, YTHDF3, an m6A reader, is considered to be a worse prognosis factor and can promote cancer progression in colorectal cancer. Mechanistically, YTHDF3 can bind m6A-modified lncGAS5, and promote the decay of lncGAS5, which can inhibit colorectal cancer progression by inhibiting YAP (Ni et al., 2019). Therefore, concomitant targeting of m6A and lncRNAs may serve as novel therapeutic targets for cancer treatment.

Herein, we explored the prognostic significance of m6A-related lncRNAs in STS using data extracted from TCGA and GTEx datasets. Based on the expression of m6A-related lncRNAs, cluster subgroups were constructed to investigate the relationship between m6A-related lncRNAs and the prognosis and immune microenvironment in STS. Furthermore, we established a novel m6A-related lncRNA-based risk signature to predict the prognosis, immune landscape, and chemosensitivity of STS patients. In conclusion, we comprehensively evaluated the roles of m6A-related lncRNAs and established a novel risk signature based on m6A-related lncRNAs that are associated with prognosis, tumor immune microenvironment, and chemotherapy efficacy in STS.

## Materials and Methods

### Patients and Datasets

The RNA sequencing (RNA-seq) data, Fragments per kilobase of transcript per Million mapped reads (FPKM) values of TCGA-SARC cohort (https://portal.gdc.cancer.gov/) and the GTEx cohort (http://commonfund.nih.gov/GTEx/), and the corresponding TCGA-SARC clinical data were downloaded from the UCSC Xena browser (https://xenabrowser.net/) (Goldman et al., 2015). For RNA-seq, the data of 263 STS tumor samples and 450 normal samples downloaded from both TCGA and GTEx were combined and normalized into log_2_(FPKM+1). A total of 259 STS patients with corresponding clinicopathological information were enrolled in our study, including 104 with leiomyosarcoma (LMS), 58 with dedifferentiated liposarcomas (DDLPS), 51 with undifferentiated pleomorphic sarcoma (UPS), 25 with myxofibrosarcomas (MFS), 10 with synovial sarcomas (SS), and 11 with other STS types. The clinical features of the STS patients are listed in Supplementary Table S1.

### Identification of m6A-Related lncRNAs

A total of 23 m6A regulators were selected based on previous studies, including writers: METTL/14/16, WTAP, VIRMA, ZC3H13, RBM15, and RBM15B; readers: YTHDC1/2, YTHDF2/3, HNRNPC, FMR1, LRPPRC, HNRNPA2B1, IGFBP1/2/3, and RBMX; and erasers: FTO and ALKBH5 (Tu et al., 2020; Xu et al., 2020; Yi et al., 2020). Genome Reference Consortium Human Build 38 (GRCh38) lncRNA annotation data were downloaded from the GENCODE website to annotate lncRNAs. Thereafter, the expression of m6A regulators was extracted based on available mRNA expression data of STS samples from TCGA-SARC. Pearson correlation analysis was conducted to identify m6A-related lncRNAs in STS samples. LncRNAs with correlation coefficients >0.4 and *p* < 0.001 were regarded as m6A-related lncRNAs.

### Consensus Clustering

Unsupervised consensus clustering method was employed to classify all STS patients into clusters according to the similarities in the expression levels of prognostic m6A-related lncRNAs by using the “ConsensusClusterPlus” R package (50 iterations and resample rate of 80%, http://www.bioconductor.org/) (Wilkerson and Hayes, 2010). Unsupervised class discovery is a technique that could detect unknown possible groups of items based on their intrinsic characteristics without external information (Wilkerson and Hayes, 2010). Consensus clustering method is a technique for investigating the number of unsupervised clusters in the data and providing quantitative and visual stability evidence (Wilkerson and Hayes, 2010). Both the elbow method and gap statistic were employed to select the optimal clustering algorithm. The optimal number of clusters was confirmed using consensus matrices and cumulative distribution functions (CDFs).

### Gene Set Enrichment Analysis

GSEA-4.0.1 software was downloaded from the website of Broad Institute (https://www.gsea-msigdb.org/gsea/index.jsp). GSEA was utilized to investigate the potential biological functions and signaling pathways related to m6A-related lncRNAs in different clusters (Du et al., 2014). Gene sets with the *p* < 0.05 were regarded as significant enrichment.

### Immune Microenvironment Assessment

Estimation of Stromal and Immune cells in Malignant Tumor tissues using Expression data (ESTIMATE), a bioinformatics method that employs gene expression signatures to evaluate the presence of infiltrating stromal and immune cells in tumor samples, was employed to calculate the ESTIMATE, stroma, and immune scores of each STS patient in TCGA using the “estimate” R package (Yoshihara et al., 2013). The infiltration of 22 immune cell subtypes was analyzed using the CIBERSORT analytical tool (Chen et al., 2018). CIBERSORT is a bioinformatics tool that can quantify the cell composition of tissue samples from their gene expression profiles (Chen et al., 2018). Spearman correlation analysis was used to investigate the relationship between the risk score and immune cell infiltration.

### Construction and Validation of Risk Signature

The 259 STS patients were randomly divided into the training cohort and the validation cohort at a ratio of 1:1 using “caret” in R. The risk signature was constructed in the training cohort. Of note, external validation in an independent validation cohort was critical for evaluating the feasibility of the risk signature. To minimize the risk of overfitting, least absolute shrinkage and selection operator (LASSO) Cox regression analysis with 10-fold cross validation and a *p* value of 0.05 was conducted to select prognosis-specific m6A-related lncRNAs for the establishment of the risk model using the R package “glmnet.” The penalty parameter (*λ*) for the risk model was confirmed by 10-fold cross-validation according to the minimum criteria. The risk scores of all patients were calculated based on the gene expression level and corresponding regression coefficients using the following formula: risk score = (0.249831693248809 × LINC01976) + (−0.489765449657647 × LINC02447) + (0.111963559093837 × SNHG1) + (0.273968944541039 × AL031985.3) + (−0.482982387224094 × AC087645.2) + (0.41981866509815 × AP005899.1) + (0.470058939980631 × YEATS2.AS1). Patients were divided into the training and validation cohorts, and divided into high-risk and low-risk groups based on the median value of risk scores of patients in the training cohort. Univariate and multivariate Cox regression analyses were conducted to verify the independent prognostic value of the risk signature.

### Investigation of the Significance of the Risk Signature in Predicting Chemosensitivity

The half inhibitory centration (IC50) of chemotherapeutic drugs was calculated to evaluate the effectiveness of the risk signature in predicting chemosensitivity in STS. The investigation was conducted and the results were visualized through “pRRophetic” and “ggplot2” R packages.

### Statistical Analysis

All the data were analyzed by R programming language 4.0.2. The differences in overall survival (OS) between grouped patients were assessed by Kaplan–Meier survival curves and log-rank analysis. The predictive performance of the constructed risk model was investigated by time-dependent ROC curve analysis through “survivalROC” R package (Blanche et al., 2013). Subgroups were analyzed to evaluate the stability of the risk signature in different groups. The differences between subgroups were compared by Wilcoxon signed-rank test and Student’s t-test. A *p* value <0.05 was statistically significant.

## Results

### Identification of m6A-Related lncRNAs in Soft Tissue Sarcomas

**FIGURE 1:**
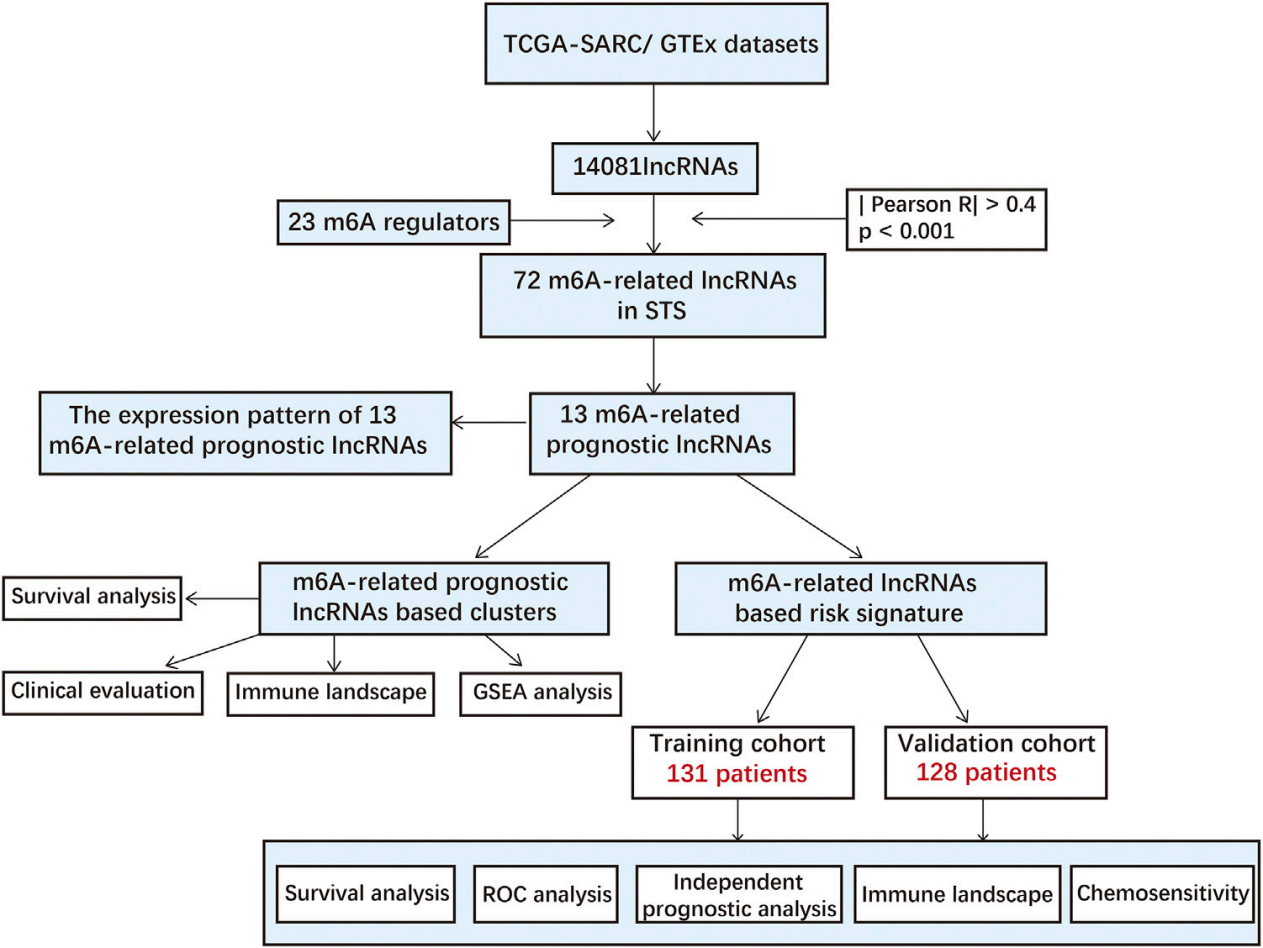
Flow diagram of this study.

**FIGURE 2:**
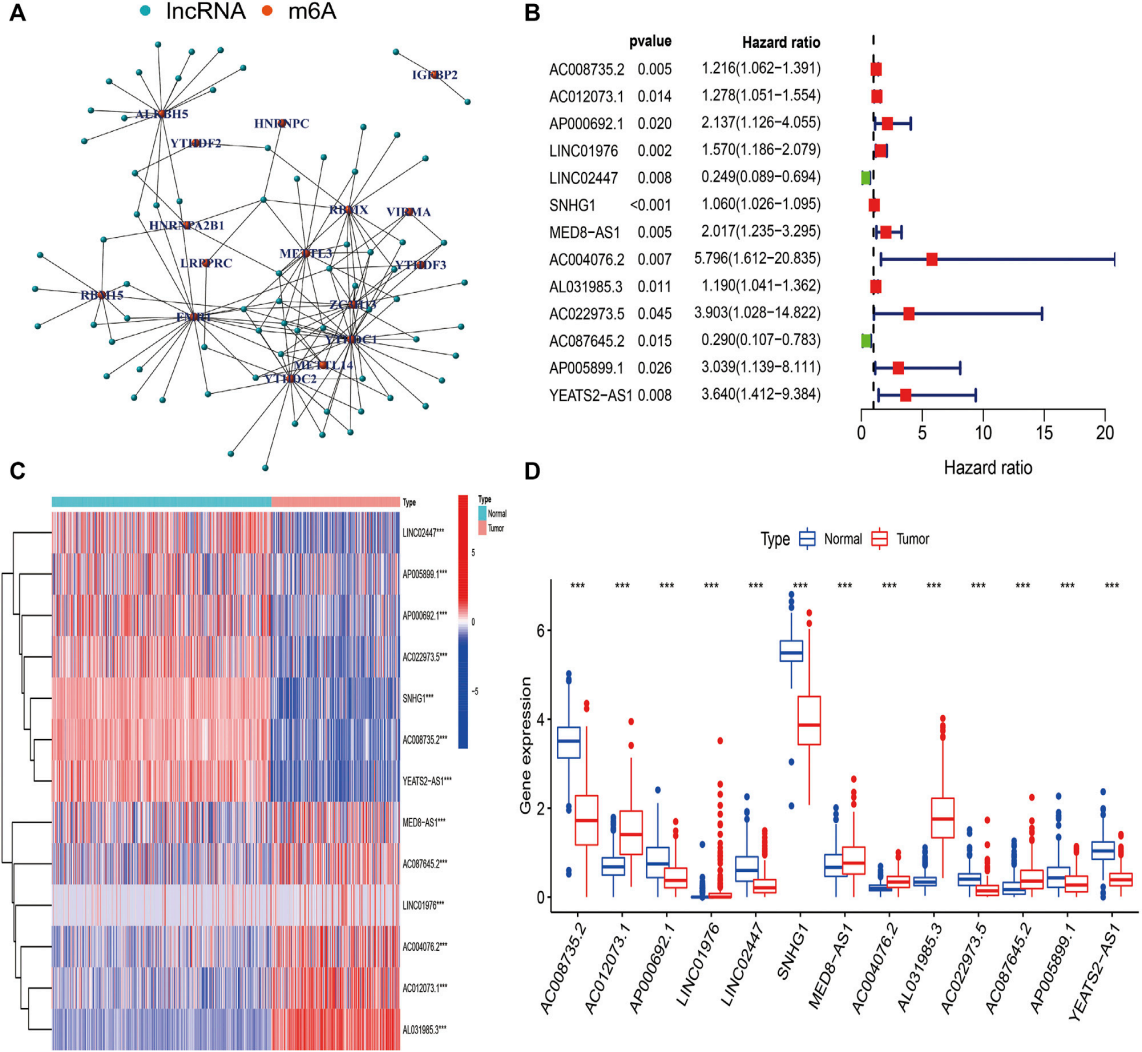
Identification of m6A-related lncRNAs in STS. **(A)** The network of 72 m6A-related lncRNAs. **(B)** The forest plot of 13 prognostic m6A-related lncRNAs. **(C)** The heatmap of 13 prognostic m6A-related lncRNAs in both tumor and normal samples. **(D)** The box plot of 13 prognostic m6A-related lncRNAs in both tumor and normal samples.

The detailed process used for the investigation in this study is presented in Figure 1. First, files were downloaded from the “GENCODE” website, and 14081 lncRNAs were extracted from TCGA-SARC and GTEx datasets for analysis. Based on previous publications, 23 m6A regulators were enrolled in our study, and the expression profiles of these m6A regulators in STS samples were extracted. Pearson correlation analysis was then carried out to select m6A-related lncRNAs in STS. lncRNAs that were significantly associated with these m6A-related regulators (coefficient >0.4 and *p* < 0.001) were confirmed as m6A-related lncRNAs. Consequently, 72 m6A-related lncRNAs were identified in STS (Supplementary Table S2). Univariate Cox regression analysis was also conducted to identify prognosis-specific m6A-related lncRNAs based on 72 m6A-related lncRNAs. As a result, 13 m6A-related lncRNAs were found to be remarkably related to the prognosis of patients with STS. An m6A-related lncRNA network, including 72 lncRNAs and 16 m6A-related regulators, is shown in Figure 2A. The forest plot revealed a hazard ratio (HR) with a 95% confidence interval (CI) for 13 m6A-related lncRNAs (Figure 2B). In addition, the expression profiles of 13 prognostic m6A-related lncRNAs were examined in 263 tumor samples and 450 normal samples. The results revealed that the expression level of seven prognostic m6A-related lncRNAs was markedly higher whereas that of six prognostic m6A-related lncRNAs was significantly lower in normal samples than in STS tumor samples (*p* < 0.001) (Figures 2C,D).

### Comprehensive Investigation of m6A-Related lncRNAs-Based Clusters

**FIGURE 3:**
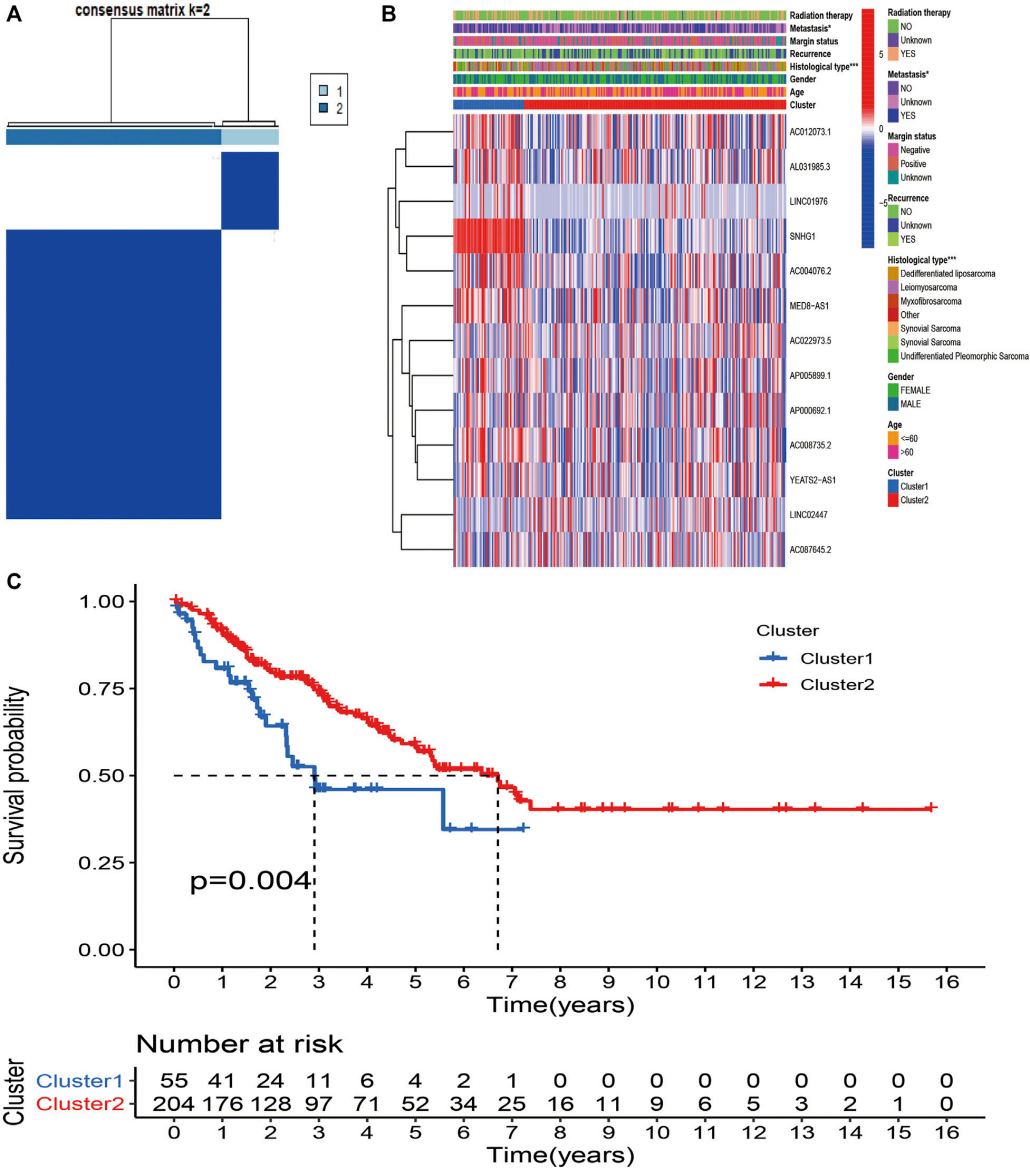
Consensus Clustering based on m6A-related lncRNAs in STS. **(A)** Consensus clustering matrix for k = 2. **(B)** Heatmap and clinicopathologic features of the two clusters (cluster1/2). **(C)** Kaplan–Meier analysis of patients in cluster 1 and cluster 2 subgroups.

To further explore the biological and clinical heterogeneity related to m6A-related lncRNAs, and whether m6A-related lncRNAs presented discernible patterns in STS, unsupervised clustering methods were utilized to classify all STS patients based on similarities in the expression patterns of 13 prognostic m6A-related lncRNAs. The optimal number of clusters (k = 2) was confirmed with optimal clustering stability k = 2–9 by combining the similarity displayed by the expression levels of prognostic m6A-related lncRNAs and the proportion of ambiguous clustering measures (Figure 3A; Supplementary Figure S1). The aim of consensus matrix (CM) plot is to evaluate the classification effect between clusters by finding the “cleanest” cluster partition. The empirical CDF plot displaying clusters k = 2–9, is aimed to find the k at which the distribution reaches an approximate maximum, indicative of the maximum stability. The delta area plot with the delta area score (*y*-axis) shows the relative increase in cluster stability. Together, a total of 259 patients with STS were divided into clusters 1 (*n* = 54) and 2 (*n* = 205). The heatmap showed the differential expression of 13 m6A-related lncRNAs between clusters, and the expression level of 13 m6A-related lncRNAs was generally higher in cluster 1 than in cluster 2. We sought to determine whether there was a distinction between the clinicopathological characteristics by cluster. Accordingly, there were significant differences in metastasis (*p* < 0.05) and histological type (*p* < 0.001) between cluster 1 and 2, indicating the potential correlations between clinical characteristics and m6A-related lncRNAs (Figure 3B). To further investigate the differences in the prognosis of patients between clusters, we conducted survival analysis. The results revealed that the survival time of patients in cluster 1 was remarkably shorter than that of patients in cluster 2 (*p* = 0.004) (Figure 3C).

**FIGURE 4:**
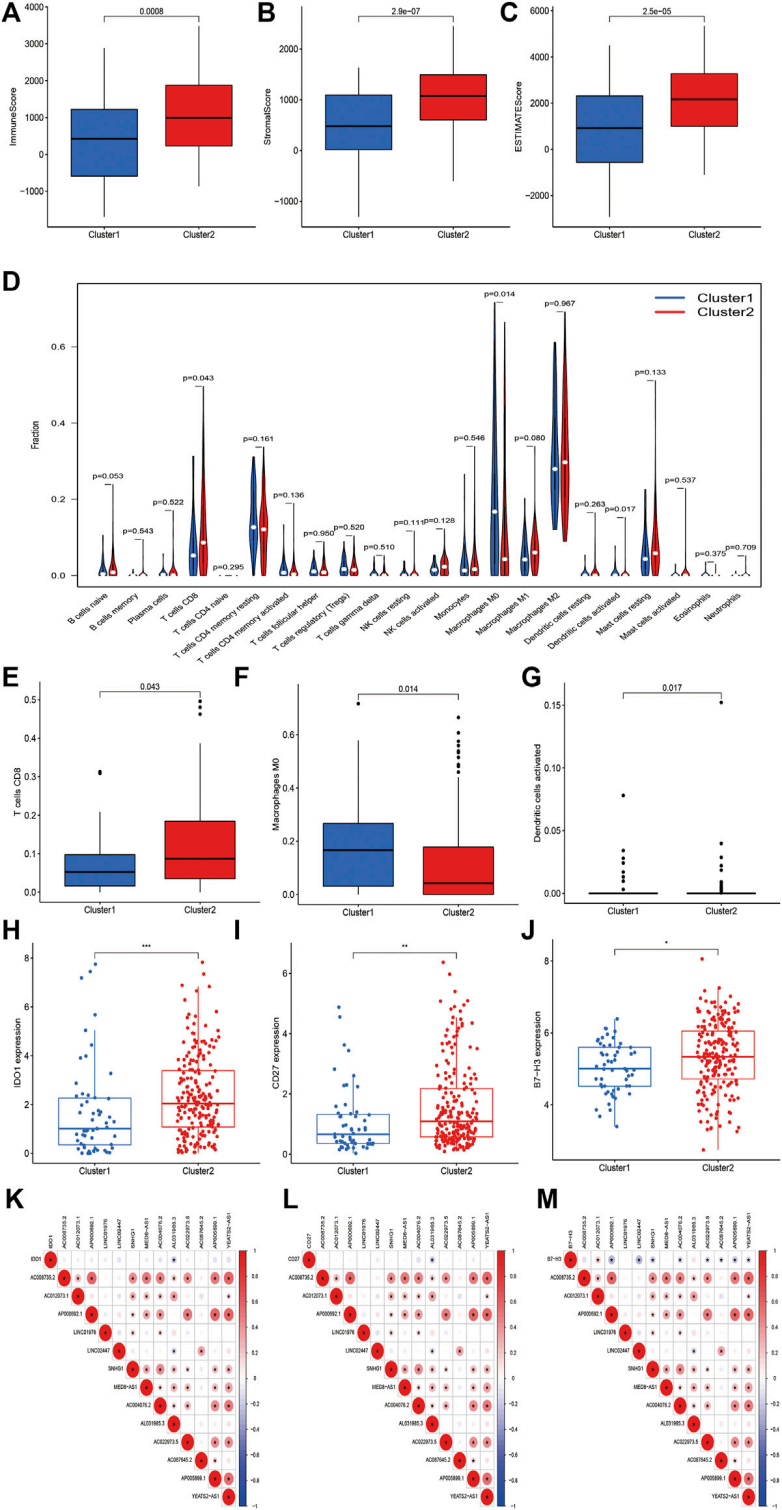
m6A-related lncRNAs were correlated with immune landscape in STS. **(A–C)** Immune, stroma and ESTIMATE scores in cluster 1 and cluster 2 subgroups. **(D)** The abundance of 22 immune cell types in cluster 1 and cluster 2 subgroups. The abundance of **(E)** CD8+ T cell, **(F)** macrophage M0 and **(G)** dendritic cell activated in cluster 1 and cluster 2 subgroups. **(H–J)** The expression of immune checkpoints **(H)** IDO1, **(I)** B7-H3 and **(J)** CD27 in cluster 1 and cluster 2 subgroups. **(K–M)** Co-expression analysis of immune checkpoints and 13 m6A-related lncRNAs.

We further investigated the association between m6A-related lncRNAs and the immune characteristics in STS. The results of Kruskal–Wallis tests revealed that immune (*p* < 0.001), stromal (*p* < 0.0001), and ESTIMATE (*p* < 0.0001) scores were markedly higher in cluster 2 than in cluster 1 (Figures 4A–C). In addition, the abundance of 22 infiltrating immune cells in the two clusters was analyzed to evaluate the relationship between m6A-related lncRNAs and infiltrating immune cells. Significant correlations were found between the proportion of infiltration of three immune cells, including activated dendritic cells (*p* = 0.017), CD8^+^ T cells (*p* = 0.043), and M_0_ macrophages (*p* = 0.014), and m6A-related lncRNA-based clusters. The abundance of activated dendritic cells and CD8^+^ T cells was significantly higher whereas that of macrophages M0 was lower in cluster 2 than in cluster 1 (Figures 4D–G). Meanwhile, the expression levels of several immune checkpoints, including IDO-1 (*p* < 0.001), CD27 (*p* < 0.01), and B7-H3 (*p* < 0.05), were markedly higher in cluster 2 than in cluster 1 (Figures 4H–J). We determined the correlations between every m6A-related lncRNA and these immune checkpoints. Accordingly, AL031985.3 was found to be negatively correlated with IDO-1 and CD27; AP000692.1, LINC02447, SNHG1, AC004076.2, AC022973.5, AC087645.2 AP005899.1, and YEATS2-AS1 were negatively correlated with B7-H3; and AC012073.1 was positively correlated with B7-H3. Moreover, the correlations between the expression of most m6A-related lncRNAs were positive, except for the negative association between LINC02447 and AL031985.3 (Figures 4K–M). In summary, these outcomes suggest that there are significant associations between m6A-related lncRNAs and the prognosis and tumor immune landscape in STS.

### Gene Set Enrichment Analysis Analysis of m6A-Related lncRNAs-Based Clusters

**FIGURE 5:**
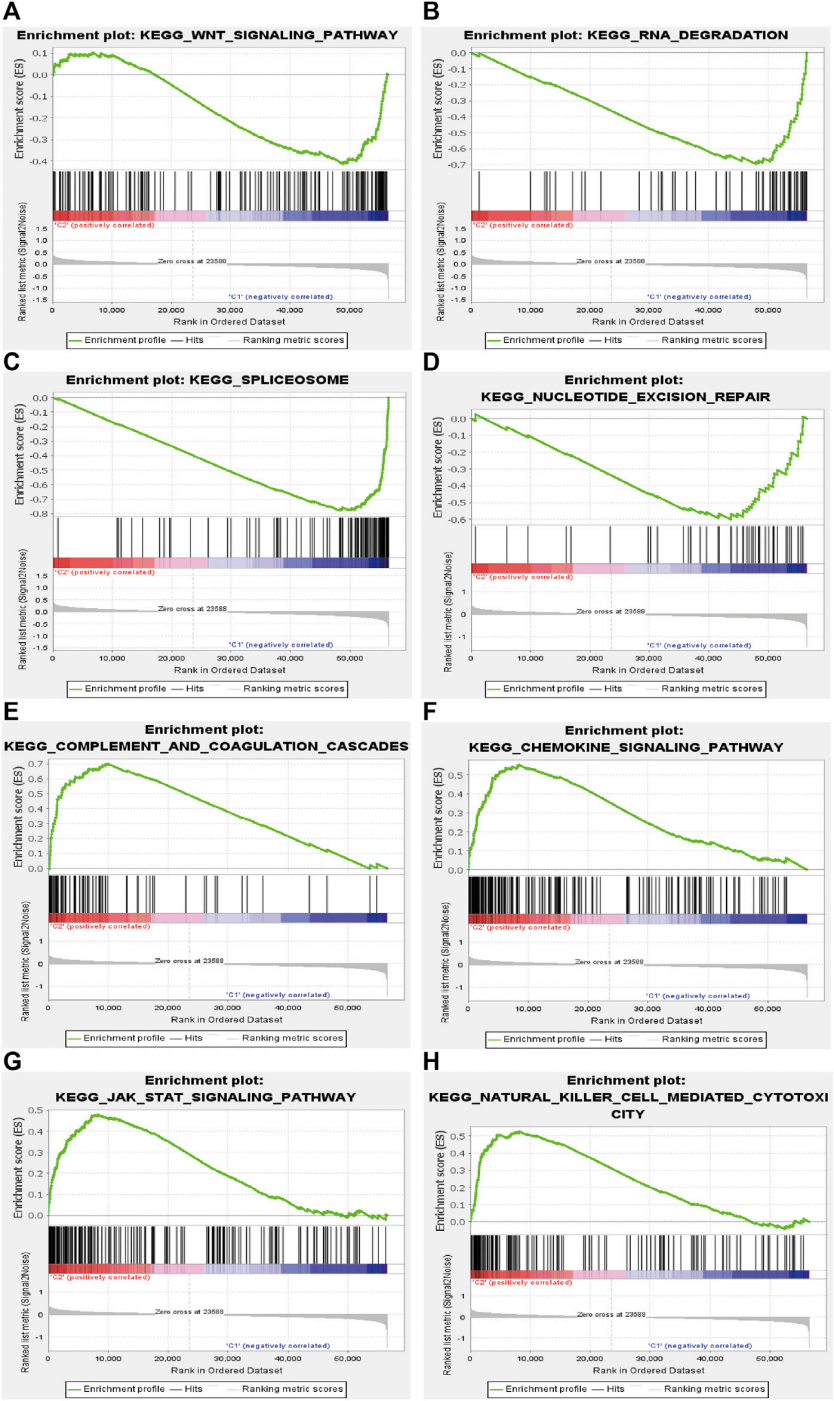
Gene set enrichment analysis (GSEA) in cluster 1 and cluster 2. **(A)** WNT signaling pathway, **(B)** RNA degradation, **(C)** spliceosome, and **(D)** nucleotide excision repair were enriched in cluster 1. **(E)** complement and coagulation cascades, **(F)** chemokine signaling pathway, **(G)** JAK/STAT signaling pathway and **(H)** NK cell mediated cytotoxicity were enriched in cluster 2.

To further investigate the potential functions of m6A-related lncRNAs, GSEA was conducted by using data from TCGA database in different clusters. The results showed that multiple signaling pathways and cellular processes such as WNT signaling pathway (Nes = 1.62, *p* = 0.015), RNA degradation (Nes = 2.09, *p* = 0.000), spliceosome (Nes = 2.24, *p* = 0.000), and nucleotide excision repair (Nes = 1.74, *p* = 0.015) were remarkably enriched in cluster 1, and chemokine signaling pathway (Nes = 1.86, *p* = 0.002), complement and coagulation cascades (Nes = 1.99, *p* = 0.000), JAK/STAT signaling pathway (Nes = 1.70, *p* = 0.008) and natural killer (NK) cell mediated cytotoxicity (Nes = 1.66, *p* = 0.038) were more enriched in cluster 2 (Figure 5). These findings provided insights into the potential biological processes and signaling pathways modulated by m6A-related lncRNAs in STS.

### Construction of m6A-Related lncRNAs-Based Risk Signature

**FIGURE 6:**
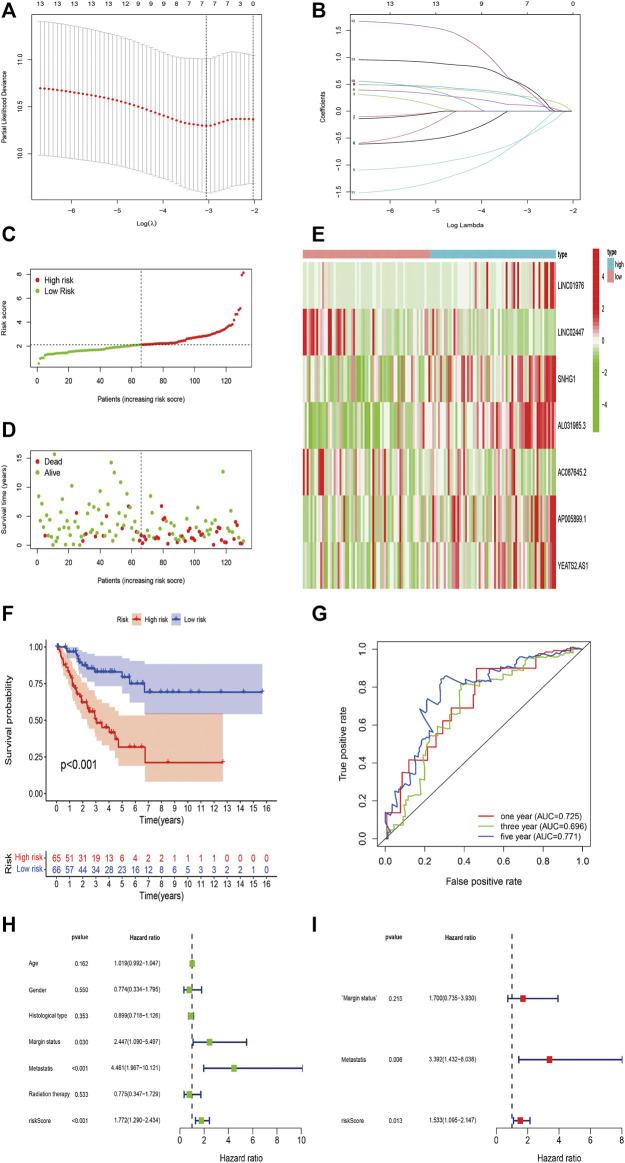
Construction of m6A-Related lncRNAs-Based Risk Signature in STS. **(A,B)** LASSO analysis with minimal lambda value. **(C,D)** Risk score and survival status of each patient in the training cohort. **(E)** Heatmap of 7 m6A-related lncRNAs in the training cohort. **(F)** Kaplan–Meier analysis of patients in the high risk and low risk groups in the training cohort. **(G)** Time-dependent ROC analysis of risk score in predicting prognoses. **(H,I)** Univariate and multivariate Cox analyses of the risk score and clinical variables in the training cohort.

**FIGURE 7:**
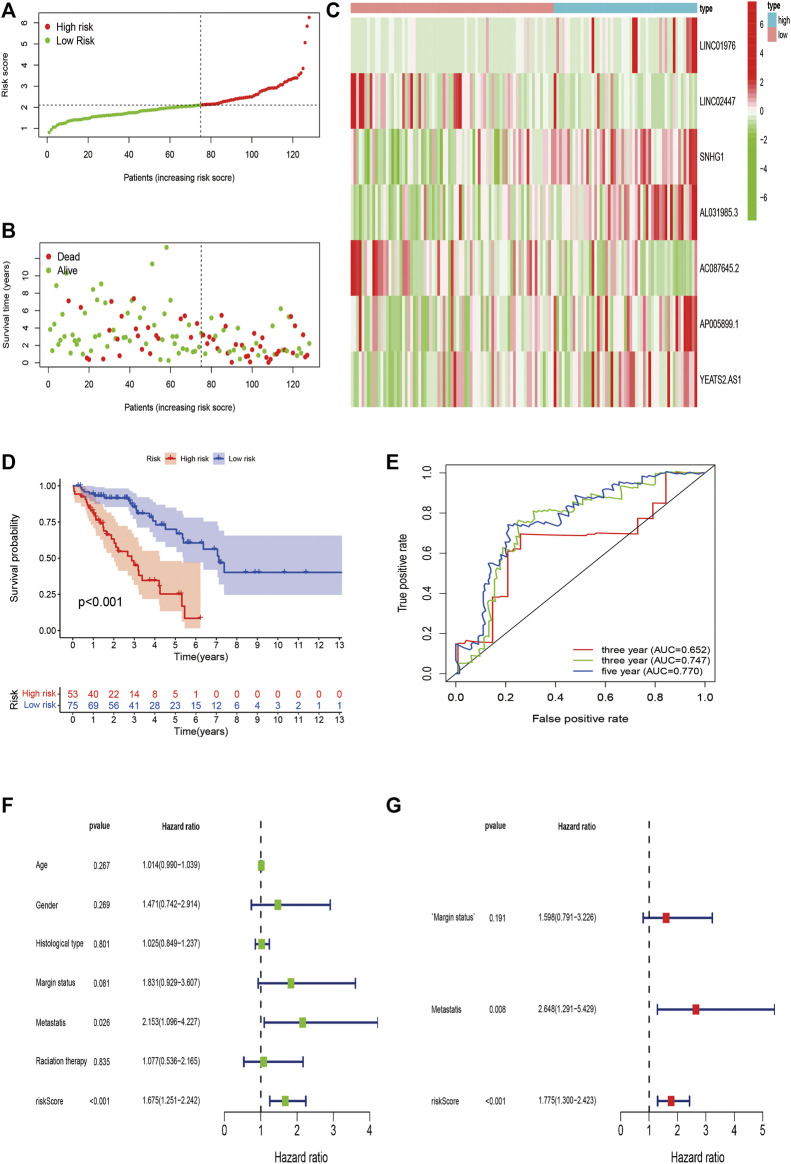
Validation of m6A-Related lncRNAs-Based Risk Signature in STS. **(A,B)** Risk score and survival status of each patient in the training validation cohort. **(C)** Heatmap of 7 m6A-related lncRNAs in the validation cohort. **(D)** Kaplan–Meier analysis of patients in the high risk and low risk groups in the validation cohort. **(E)** Time-dependent ROC analysis of risk score in predicting prognoses. **(F,G)** Univariate and multivariate Cox analyses of the risk score and clinical variables in the validation cohort.

To further investigate the predictive value of m6A-related lncRNAs in STS, we established a novel risk signature on the basis of m6A-related lncRNAs by LASSO Cox regression algorithm. All patients were randomly divided into two cohorts, including 131 in the training cohort and 128 in the validation cohort. LASSO regression analysis revealed 7 m6A-related lncRNAs with the minimum lambda value in the training cohort (Figures 6A,B). The risk score of each patient was calculated, and then patients in both cohorts were distinguished into the high-risk and low-risk subgroups by the median value of risk scores in the training cohort. Additionally, the risk plot indicated that higher risk scores were closely related to shorter survival time and worse survival status in STS (Figures 6C,D). The heatmap revealed the different expression patterns of 7 m6A-related lncRNAs between the high-risk and low-risk groups (Figure 6E). The Kaplan–Meier survival curve indicated that high-risk STS patients had a worse OS in comparison with low-risk patients (*p* < 0.001) (Figure 6F). Besides, the time-dependent ROC curve identified the excellent performance of this risk signature in predicting OS of STS patients. The area under ROC curve (AUC) of the risk signature for 1-/3-/5-year OS was 0.735, 0.696 and 0.771 respectively (Figure 6G). Furthermore, we conducted univariate and multivariate analyses to identify the capability of the risk signature as an independent indicator for the prognosis in STS. Univariate Cox regression analysis results reveled that m6A-related lncRNAs risk model was remarkably connected with the prognosis of STS patients (HR = 1.772, 95% CI = 1.290–2.434; *p* < 0.001) (Figure 6H). Furthermore, multivariate Cox regression analysis indicated that m6A-related lncRNAs risk model could function as an independent prognostic predictor (HR = 1.533, 95% CI = 1.095–2.147; *p* = 0.013) (Figure 7I). Additionally, univariate analysis indicated that margin status and metastasis were also linked with the prognosis of STS patients, and multivariate analysis confirmed that metastasis presented as an independent prognosis-related variable. Together, these results indicated that the m6A-related lncRNAs-based risk signature had a robust and stable OS-predictive ability for STS patients.

### Validation of m6A-Related lncRNAs-Based Risk Signature

**FIGURE 8:**
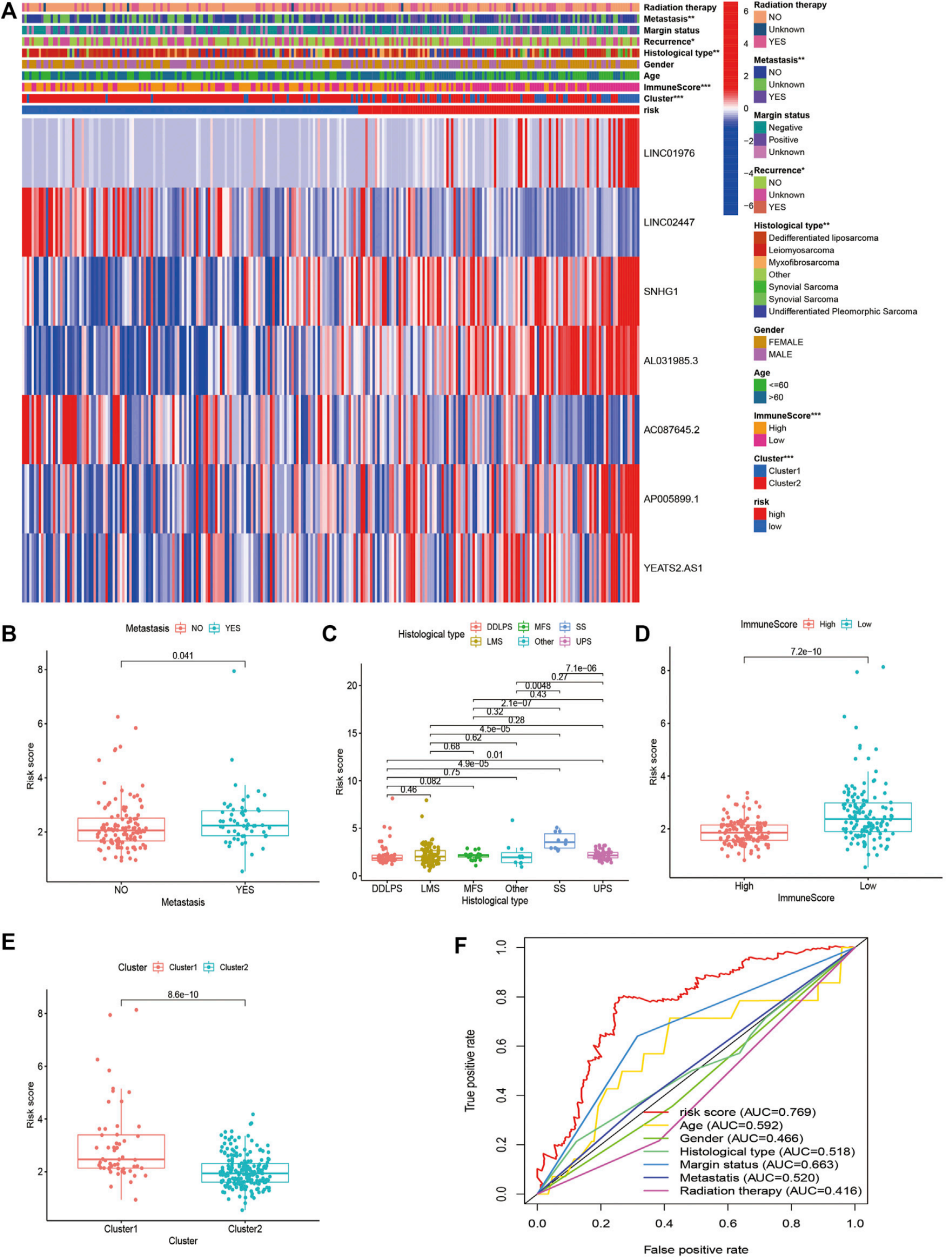
Clinical Evaluation of m6A-Related lncRNAs-Based Risk Signature in STS. **(A)** The heatmap of the associations between the risk score and clinicopathological features. **(B–E)** The boxplots of the associations between the risk score and **(B)** metastasis, **(C)** histological type, **(D)** immune score, and **(E)** cluster. **(F)** A comparison of 5-year ROC curve with other clinical characteristics.

We validated the predictive value of the risk signature in an independent validation cohort. The risk score plot and survival status plot of STS patients showed that the survival time and survival rate decreased with increasing risk score (Figures 7A,B). We also evaluated the differential m6A-related lncRNA expression between the high-risk and low-risk groups (Figure 7C). Survival analysis indicated that the OS of high-risk patients was remarkably worse than that of their low-risk counterparts (*p* < 0.001) (Figure 7D). The time-dependent ROC curve was displayed in Figure 8E, and the 1-/3-/5-year AUC was 0.652, 0.747, and 0.770, respectively, indicating the good predictive value of the risk signature. Univariate analysis also revealed a significant association between the risk score and prognosis (HR = 1.675, 95% CI = 1.251–2.242; *p* < 0.001), and multivariate analysis further confirmed the role of the risk score as an independent prognostic predictor in STS (HR = 1.775, 95% CI = 1.300–2.423; *p* < 0.001) (Figures 7F,G). In conclusion, these findings align with the results of the training cohort, thereby verifying that the risk signature are robust prognostic biomarkers in STS.

### Clinical Evaluation of Risk Signature

**FIGURE 9:**
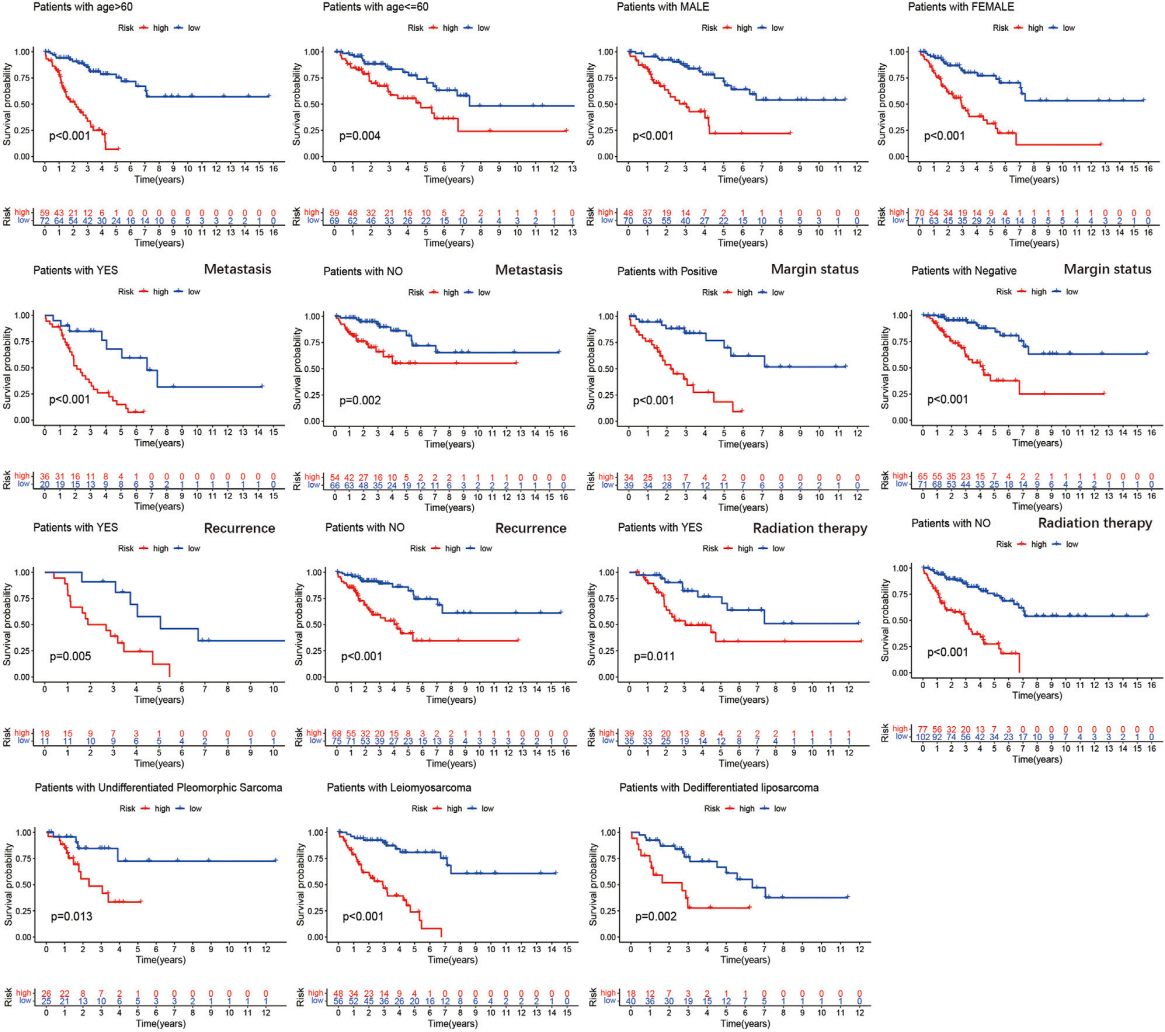
Kaplan–Meier analysis of the association between the risk signature and overall survival in subgroups based on age, gender, confirmed recurrence, confirmed metastasis, confirmed radiation therapy, margin status, and histological type.

To further verify the prognostic value of the m6A-related lncRNA-based risk signature in STS, we conducted a survival analysis on the risk signature of patients in diverse subgroups based on age, sex, confirmed recurrence, confirmed metastasis, confirmed radiation therapy, margin status, and histological type. High-risk patients were confirmed to have a worse prognosis for both male and female patients, regardless of whether they were ≤60 or >60 years old, with or without metastasis, with positive margin status or negative margin status, with or without radiation therapy, with or without recurrence, and whether they were patients with DDLPS, LMS, and UPS (Figure 8). These results verified the promising role of the m6A-related lncRNA-based risk signature as a prognostic predictor for STS patients, regardless of clinical factors. The relationship between the risk signature and clinicopathological parameters of STS was also explored. The risk score was found to be significantly correlated with metastasis, margin status, histological type, immune score, and clusters of m6A-related lncRNAs (Figures 9A–E). The risk score of patients with metastasis was higher than that of patients without metastasis (*p* = 0.041); patients with synovial sarcoma had a remarkably higher risk score than those with DDLPS (*p* < 0.001), LMS (*p* < 0.001), MFS (*p* < 0.001), and UPS (*p* < 0.001). Patients with high immune scores presented a lower risk score than those with low immune scores (*p* < 0.001); and patients in cluster 1, who exhibited poor OS, presented higher risk scores than those in cluster 2 (*p* < 0.001). By comparing the 5-year AUC of the risk signature with other clinical characteristics, we found that the risk signature had a significantly higher AUC than other clinical parameters (Figure 9F).

### Risk Signature was Correlated With the Immune Landscape

**FIGURE 10:**
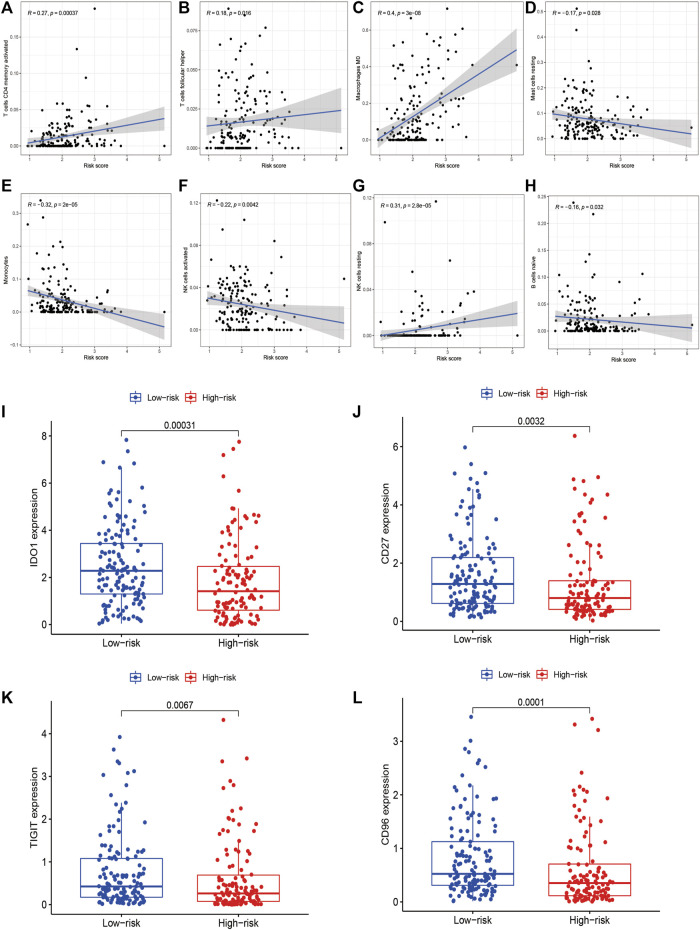
The correlations between the risk signature and immune microenvironment in STS. **(A–H)** The correlations between the risk score and immune cells infiltration. The risk score was positively related to **(A)** activated memory CD4^+^ T cells, **(B)** follicular helper T cells, **(C)** resting NK cells, and **(D)** M0 macrophages, while negatively related to **(E)** activated NK cells, **(F)** resting mast cell, **(G)** monocytes, and **(H)** naïve B cells. **(I–L)** The correlations between the risk signature and the expression of immune checkpoint **(I)** IDO1, **(J)** CD96, **(K)** TIGIT and **(L)** CD27.

With regards to immune microenvironment of STS, the risk score was positively related to activated memory CD4^+^ T cells (R = 0.27, *p* < 0.0001), follicular helper T cells (R = 0.18, *p* = 0.016), resting NK cells (R = 0.31, *p* < 0.0001), and M0 macrophages (R = 0.4, *p* < 0.0001), while negatively related to activated NK cells (R = -0.22, *p* = 0.0042), resting mast cell (R = -0.17, *p* = 0.028), monocytes (R = -0.32, *p* < 0.0001), and naïve B cells (R = -0.16, *p* = 0.032) (Figures 10A–H). Besides, the expression level of several immune checkpoints, including IDO-1 (*p* < 0.001), CD96 (*p* = 0.0032) TIGIT (*p* = 0.0067), and CD27 (*p* < 0.001) was markedly higher in the low-risk group in comparison to the high-risk group (Figures 10I–L). These findings confirmed the associations between the risk signature and immune landscape in STS.

### Risk Signature was Correlated With Chemosensitivity

**FIGURE 11:**
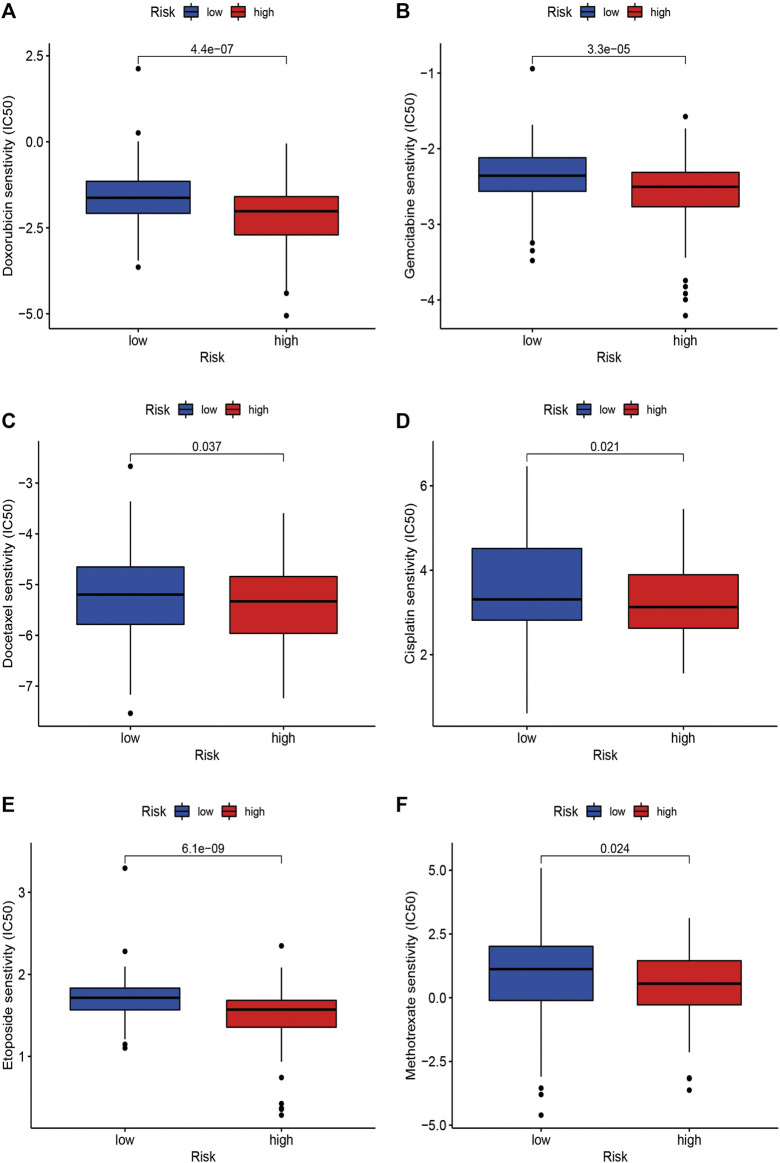
The correlations between the risk signature and chemosensitivity in STS. The IC50 of **(A)** doxorubicin, **(B)** gemcitabine, **(C)** docetaxel, **(D)** etoposide, **(E)** cisplatin, and **(F)** methotrexate in the high-risk and low-risk groups.

As for the chemotherapeutic efficacy, we attempted to assess the potential role of the risk model as a chemosensitivity predictor in STS in clinic. The results revealed that several chemotherapeutic agents in low-risk patients had higher IC50, including doxorubicin (*p* < 0.001), gemcitabine (*p* < 0.001), cisplatin (*p* = 0.021), etoposide (*p* < 0.001), and methotrexate (*p* = 0.024), suggesting that low-risk patients were more sensitive to these chemotherapeutic drugs (Figure 11). These findings identified the promising role of this risk signature as a predictor for chemotherapy efficacy in the treatment of STS patients.

## Discussion

In the present study, we performed a hitherto undocumented investigation of the roles of m6A-related lncRNAs and a novel m6A-related lncRNA-based risk signature in STS. First, 259 STS patients from TCGA-SARC dataset were enrolled in our study. We identified 72 lncRNAs that were closely related to 16 m6A-related regulators and constructed the m6A-related lncRNA network. Among the 72 m6A-related lncRNAs, 13 m6A-related lncRNAs were further identified to have significant prognostic significance for STS patients in TCGA-SARC dataset. Thereafter, we identified two clusters of STS patients based on prognostic m6A-related lncRNAs by consensus clustering to clarify whether m6A-related lncRNAs could affect the prognosis and immune landscape of STS. Based on the survival analysis, patients in cluster 1 had worse OS than those in cluster 2, indicating that m6A-related lncRNAs were correlated with prognosis of patients with STS, and might be gainfully employed to serve as an independent prognosis-specific biomarker in STS. Furthermore, patients in cluster 2 had a higher immune score, stroma score, and ESTIMATE score and higher expression levels of several immune checkpoints, including IDO1, CD27, and B7-H3, than patients in cluster 1. The expression of several m6A-related lncRNAs, such as AL031985.3, SNHG1, and YEATS2-AS1, was also found to be closely related to the expression of these immune checkpoints, indicating the potential role of m6A-related lncRNAs as modulators of immune checkpoints. GSEA was performed to determine the potential biological functions and signaling pathways modulated by m6A-related lncRNAs, which contribute to the molecular heterogeneity between clusters. GSEA indicated that immune-related signaling pathways, such as the JAK-STAT signaling pathway, chemokine action signaling pathway, NK cell-related signaling pathway, and complement and coagulation cascade-related processes, were more enriched in cluster 2 than in cluster 1. Such findings indicate that m6A-related lncRNAs might function as critical modulators of immune-related processes during cancer progression, thereby providing a direction for further studies to explore the involvement of m6A-related lncRNAs in regulating the immune microenvironment in STS.

Among the 13 m6A-related prognostic lncRNAs, some lncRNAs have been found to play critical roles in cancer progression. For instance, SNHG1, a novel oncogenic lncRNA with abnormal expression in various cancer types, can contribute to osteosarcoma tumorigenesis and progression via complex mechanisms (Xu et al., 2018; Thin et al., 2019; Wu et al., 2021). SNHG1, which is upregulated in both osteosarcoma tissues and cells, is reported to be correlated with worse OS of osteosarcoma patients and can facilitate cell proliferation, migration and invasion via the miR-101-3p/ROCK1 axis, the miR-326/NOB1 axis, and the miR-577/WNT2B/Wnt/β-catenin axis, respectively (Jiang et al., 2018; Wang et al., 2018; Deng et al., 2019). Several studies have also revealed that AL031985.3 may serve as a promising prognostic predictor in hepatocellular carcinoma (Jia et al., 2020; Kong et al., 2020). Nevertheless, the roles of most m6A-related lncRNAs in cancer progression require further investigation.

To further evaluate the roles of m6A-related lncRNAs and facilitate the clinical applications of m6A-related lncRNAs as biomarkers and therapeutic targets in STS, we established a novel m6A-related lncRNA-based risk signature. Several studies have constructed risk signatures based on the expression of candidate genes in diverse cancers (Li et al., 2017; Hong et al., 2020; Liu et al., 2021a). The expression and coefficient of candidate genes were employed to calculate the risk scores, and patients with higher risk scores were suggested to have a worse prognosis. For instance, a recent study constructed a novel ferroptosis-related gene signature that could perfectly predict the prognosis of STS (Huang et al., 2021). Recently, several studies have established risk models based on m6A regulators for multiple cancers (Ji et al., 2020; Pan et al., 2020; Xu et al., 2020). Notably, Tu et al. found that m6A-related lncRNAs were significantly correlated with clinical outcomes and established an m6A-related lncRNA-based risk model that could effectively predict the prognosis of glioma, thereby highlighting the promising roles of m6A-related lncRNAs in human malignancies (Tu et al., 2020). However, no studies have established risk models associated with m6A-related lncRNAs in STS. To the best of our knowledge, this is the first study to establish a risk model based on m6A-related lncRNAs to explore the potential roles and clinical value of m6A-related lncRNAs in STS. The survival analysis results confirmed that the high-risk score was closely related to worse OS, and subgroup analyses further verified the prognostic significance of m6A-related lncRNAs in STS. The 5-year AUCs in both the training and validation cohorts were over 0.75, indicating that the risk signature had excellent performance and stability in predicting the prognosis of STS patients.

The tumor immune microenvironment is critically involved in cancer initiation and progression. In our study, we attempted to evaluate the relationship between the risk signature and the immune microenvironment in STS. Based on the results, patients with high immune scores had lower risk scores than those with low immune scores. Previous studies have found that STS patients with high immune scores calculated by ESTIMATE had a better prognosis, which was consistent with the results of our risk signature and cluster analysis based on m6A-related lncRNAs (Hu et al., 2020; Wang et al., 2021). In addition, a positive relationship was found between the risk score and the infiltrating proportion of M0 macrophages, activated memory CD4^+^ T cells, follicular helper T cells, and resting NK cells, whereas negative correlations were found between the risk score and the infiltration of activated NK cells, resting mast cells, monocytes, and naïve B cells. Infiltrating immune cells in the tumor immune microenvironment have been found to be closely connected with the prognoses of cancer patients. For instance, NK cells can exert anti-tumor effects by activating an antigen-independent immune response (Liu et al., 2021b). Therefore, the activation of NK cells is significantly correlated with better prognosis, while the higher levels of resting NK cells infiltration indicate a worse chance of survival in human malignancies (Bigley and Simpson, 2015; Bisheshar et al., 2020). Similarly, the infiltration of M0 macrophages is positively related to poor clinical outcomes in human malignancies, including STS, which aligns with the findings of our study (Zhu and Hou, 2020). Thus, the worse clinical outcomes of the high-risk group may be associated with infiltrating immune cellular populations, and m6A-regulated lncRNAs may function as critical modulators of immune cell infiltration in STS.

Immune suppression also plays a critical role in the initiation and progression of cancer. Cancer cells can activate immune checkpoint pathways that can inhibit anti-tumor immune functions, thereby contributing to cancer immune tolerance (Topalian et al., 2016; Abril-Rodriguez and Ribas, 2017). Hence, blocking immune checkpoints to achieve anti-tumor immunity has provided breakthroughs in cancer treatment. Several immune checkpoint inhibitors, such as PD-1/PD-L1 inhibitors, have been developed to treat several human malignancies (Darvin et al., 2018). Although immune checkpoint inhibitors have been recently employed in STS, the therapeutic efficacy and prolonged benefits are limited, which may be attributed to the indefinite expression pattern and roles of immune checkpoints in STS (Zhu et al., 2020). Therefore, we further evaluated the correlations between m6A-related lncRNAs and immune checkpoint expression, which may facilitate the application of immunotherapy in STS treatment. Our results showed that the risk score was closely related to the expression of several immune checkpoints, including IDO1, CD27, CD96, and TIGIT, which were highly expressed in the low-risk group. IDO1, a novel immune checkpoint, is a rate-limiting metabolic enzyme that can contribute to the conversion of the essential amino acid tryptophan (Trp) into kynurenines (Kyn). The upregulation of IDO1 has been detected in diverse human cancer types, and its prognostic significance in human cancers has been investigated in multiple studies. Most studies indicate that IDO1 is correlated with poor prognosis in various cancer types, such as ovarian cancer, esophageal cancer, and penile squamous cell carcinoma (Yu et al., 2018; Kiyozumi et al., 2019; Zhou et al., 2020). However, some studies have reported contradictory outcomes (Riesenberg et al., 2007). According to a previous study, IDO1 expression could serve as a favorable prognostic biomarker in undifferentiated pleomorphic sarcoma (UPS), which is consistent with our results (Ishihara et al., 2021). Our risk signature may help predict immune checkpoint expression and function as effective biomarkers for immunotherapy efficacy in STS. Owing to small sample sizes and diverse histological types compared with other cancer types, it is difficult to identify the correlations between immune checkpoints and the prognosis in STS. Studies based on large clinical samples are needed to elucidate the roles of immune checkpoints, as well as the correlations between m6A-realted lncRNAs and immune checkpoints in STS.

Currently, chemotherapy is widely used to treat STS and can prolong the survival time of patients. Commonly used chemotherapeutic drugs for STS include doxorubicin, ifosfamide, and gemcitabine (Ratan and Patel, 2016). However, the development of chemoresistance has become the main obstacle in the improvement of chemotherapeutic efficacy and prognosis of patients with STS (Lin et al., 2020). Thus, it is critical to develop effective biomarkers to predict chemosensitivity and novel therapeutic targets to reverse chemoresistance in STS. Notably, our study indicated that the m6A-related lncRNA-based risk signature could function as a promising predictor of chemosensitivity in STS. Low-risk patients were found to be more sensitive to several chemotherapeutic agents, including doxorubicin, gemcitabine, docetaxel, cisplatin, etoposide, and methotrexate. Therefore, these m6A-related lncRNA-based risk signatures may function as promising predictors of chemosensitivity in STS, and dynamic monitoring of these m6A-related lncRNAs may effectively help to evaluate the responses of STS patients to chemotherapy, thereby selecting the most suitable chemotherapy protocol for individual patients. In addition, targeting these m6A-related lncRNAs may be a promising method to enhance the chemosensitivity of STS.

Our study had several limitations. First, our findings are based on data extracted from public databases; therefore, these results should be further verified in our own cohort. Second, the interactions of lncRNAs and m6A regulators were not validated via *in vivo* and *in vitro* experiments. Third, the potential biological processes and signaling pathways modulated by m6A-related lncRNAs in STS were not confirmed. Further, more research is needed to clarify the functional targets of m6A-related lncRNAs in the pathogenesis of STS. Finally, the functional roles and mechanisms of m6A-related lncRNAs have not been investigated. Hence, further *in vivo* and *in vitro* studies and clinical investigations are warranted to illustrate the critical roles of m6A-related lncRNAs in STS.

## Conclusion

In summary, we identified that m6A-related lncRNAs could predict the prognosis and tumor immune landscape in STS. In addition, we established a novel m6A-related lncRNA-based risk signature that was remarkably linked with the prognosis and immune microenvironment, and could effectively predict the prognosis and chemotherapeutic efficacy of STS. Our findings could persuade researchers to focus on the potential roles and detailed mechanisms of m6A-related lncRNAs in STS, which would enable novel therapeutic targets for STS treatment to be found and the prognosis of STS patients to be improved.

## Data Availability

The original contributions presented in the study are included in the article/Supplementary Material, further inquiries can be directed to the corresponding author.
