# Supplementary material for: Construction of a Novel Signature and Prediction of the Immune Landscape in Soft Tissue Sarcomas Based on N6-Methylandenosine-Related LncRNAs
Source: Front Mol Biosci. 2021 Oct 15;8:715764. doi: 10.3389/fmolb.2021.715764 (PMC8559337; doi:10.3389/fmolb.2021.715764)
Supplement: Supplementary file 1 [file DataSheet1.docx]

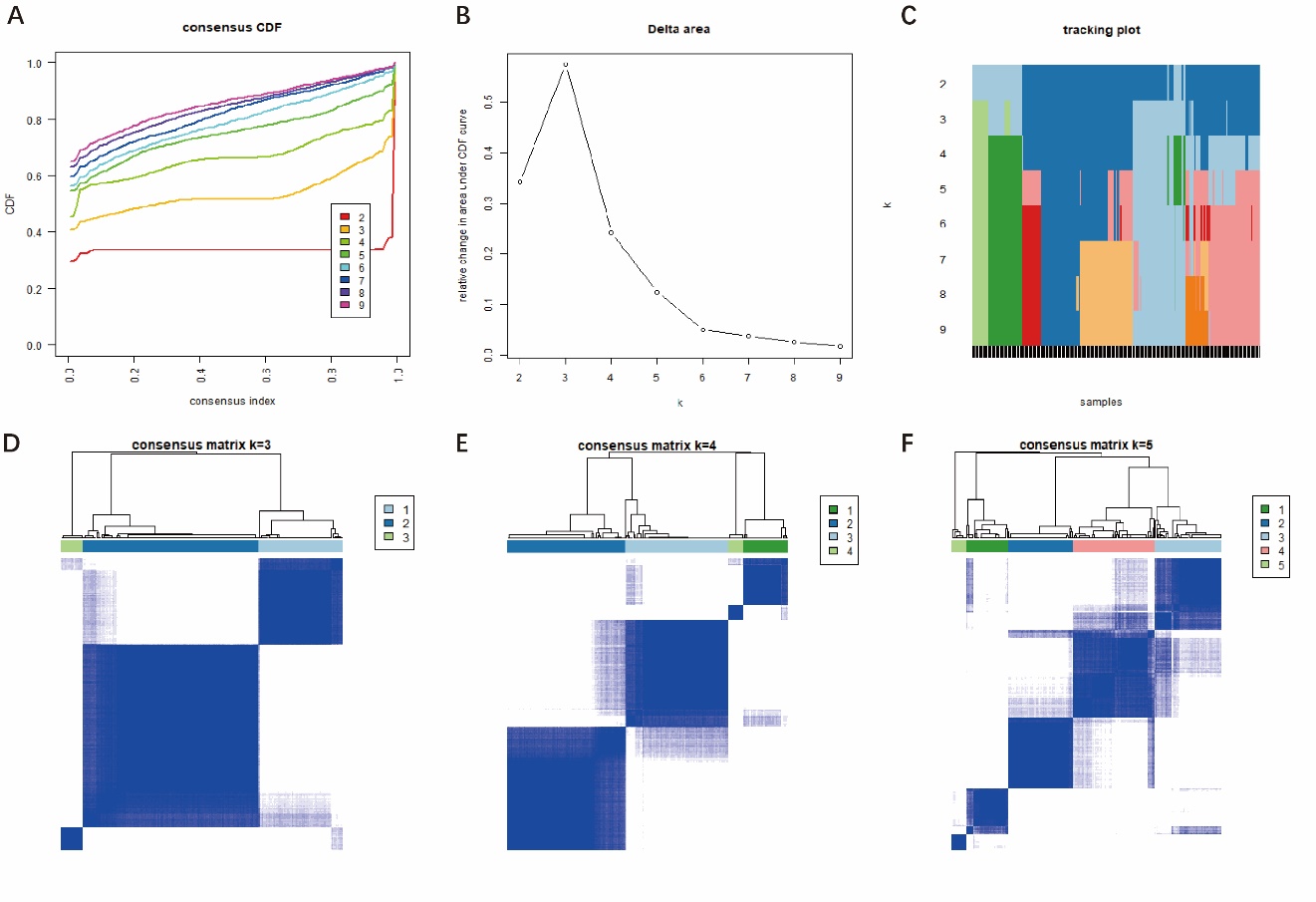


**Figure S1. Unsupervised consensus clustering by m6A-related lncRNAs. (A)** Consensus clustering cumulative distribution function (CDF) for k=2 to 9. **(B)** Relative change in area under the CDF curve for k=2 to 9. **(C)** Tracking plot for k=2 to 9. **(D-F)** Consensus clustering matrix for k=2, 3 and 4.

**Table S1** Clinical characteristics of STS patient data sets in the study

| **Characteristics (n; %)** | **TGCA-SARC (n=259)** |
| --- | --- |
| **Age** |  |
| ≤ 60 | 128 (49.4) |
| > 60 | 131 (50.6) |
| **Gender** |  |
| Male | 118 (45.6) |
| Female | 141 (54.4) |
| **Histological type** |  |
| DDLPS | 58 (22.4) |
| LMS | 104 (40.2) |
| MFS | 25 (9.6) |
| SS | 10 (3.9) |
| UPS | 51 (19.7) |
| Other | 11 (4.2) |
| **Metastasis** |  |
| Yes | 56 (21.6) |
| No | 120 (46.3) |
| Unknow | 83 (32.0) |
| **Margin status** |  |
| Positive | 73 (28.2) |
| Negative | 136 (52.4) |
| Unknow | 40 (15.4) |
| **Recurrence** |  |
| Yes | 29 (11.2) |
| No | 143 (55.2) |
| Unknow | 87 (33.6) |
| **Radiotherapy** |  |
| Yes | 74 (28.6) |
| No | 179 (69.1) |
| Unknow | 6 (2.3) |

DDLPS: dediferentiated liposarcoma, LMS: leiomyosarcoma, MFS: myxofbrosarcoma,

SS: synovial sarcoma, UPS: undiferentiated pleomorphic sarcoma
